# Editorial: Bone and Cartilage Diseases – The Role and Potential of Natural Products, Volume II

**DOI:** 10.3389/fphar.2023.1194875

**Published:** 2023-05-02

**Authors:** Ming Pei, Daohua Xu, Longhuo Wu

**Affiliations:** ^1^ Stem Cell and Tissue Engineering Laboratory, Department of Orthopaedics, West Virginia University, Morgantown, WV, United States; ^2^ Guangdong Key Laboratory for Research and Development of Natural Drugs, School of Pharmacy, Guangdong Medical University, Dongguan, China; ^3^ College of Pharmacy, Gannan Medical University, Ganzhou, China

**Keywords:** osteoporosis, natural products, traditional Chinese medicine, genistein, natural alkaloids, dehydromiltirone, kidney-tonifying Chinese herbs, bone and cartilage diseases

As a bone and cartilage disease, osteoporosis, resulting from an imbalance in remodeling between bone formation and resorption, has become a major global public concern, often resulting in fracture and seriously impacting activities of daily living (Hernlund et al., 2013). Evidence shows that osteoporosis increases the severity of cartilage damage via subchondral bone (Calvo et al., 2007; Wada et al., 2023); thus, the treatment of osteoporosis also benefits cartilage health. Due to sex differences, females have a higher risk of developing osteoporosis than males (Patel et al., 2023). Common anti-osteoporotic pharmaceuticals are effective in the prophylaxis and treatment of osteoporosis; however, various side effects resulting from western medicines drive many women to seek botanicals as an alternative therapy (Słupski et al., 2021). Traditional Chinese medicine (TCM) is an excellent candidate for effective treatment with fewer side effects. In this work, five articles are included to discuss kidney-tonifying TCM (review), kidney-tonifying Chinese herbs (review), genistein (review), natural alkaloids (review), and dehydromiltirone (original study) for potential treatment of osteochondral diseases, particularly for osteoporosis.

Based on the TCM theory that “kidney governs bone,” kidneys can be a target for the treatment of bone diseases using “kidney-tonifying Chinese herbs” and “kidney-tonifying TCM (KTTCM).” In a review paper by Duan et al., they summarized KTTCM’s ability to regulate estrogen expression, promote osteoblasts, inhibit osteoclasts, and restrain adipogenesis, as well as related molecular mechanisms including but not limited to osteoprotegerin (OPG)/receptor activator of nuclear factor kappa B (RANK)/RANK ligand (RANKL), bone morphogenetic protein (BMP)/suppressor of mothers against decapentaplegic (Smad), mitogen-activated protein kinases (MAPKs), and wingless/integrated (Wnt) signaling pathways. Furthermore, Jiao et al. reviewed the mechanisms by which kidney-tonifying Chinese herbs prevent osteoclastogenesis, emphasizing their immune effect.

Genistein, a polyphenolic isoflavone, is found in varying dietary vegetables, such as soy beans and leguminous plants; it exhibits biological effects, such as anti-cancer, anti-inflammation, anti-oxidation, and bone/cartilage protection. The similarity in structure to estrogen provides genistein with estrogen-like activity in defending against osteoporosis and osteoarthritis. Wu and Liu summarized what is known about genistein in the treatment of bone and cartilage diseases, including intervertebral discs, osteoporosis, osteoarthritis, and rheumatoid arthritis. The involved molecular pathways were also discussed in each section to provide in-depth insights for future treatment. Ultimately, the authors also discussed clinical perspectives and limitations, such as low water solubility and the poor bioavailability of genistein, which need to be further investigated to improve genistein’s curative effect.

Natural alkaloids, a class of naturally occurring organic nitrogen-carrying bases, have diverse and crucial physiological effects on humans; this class includes strychnine, morphine, quinine, ephedrine, and nicotine. Additional evidence shows that alkaloids play a role in bone and cartilage diseases. Lin et al. reviewed the effect of natural alkaloids on mesenchymal stem cells, osteoblasts, osteoclasts, and related signaling pathways; their evidence supported the beneficial effects of alkaloids in promoting osteoblast proliferation and inhibiting osteoclast formation. Current efforts indicate that alkaloids could be a novel solution for the treatment of osteoporosis.

Dehydromiltirone (DHT), a diterpenoid quinone originating in TCM, *Salvia miltiorrhiza Bge,* and *Salvia przewalskii Maxim*, have been widely used to treat some diseases, such as liver diseases (Yue et al., 2014). Using the approach of network pharmacology and cellular studies, Deng et al. designed an interesting experiment to examine the potential mechanism of DHT in the treatment of osteoporosis. They found that docking analysis supported that DHT bound stably to MAPK14 more than other proteins, suggesting that DHT may influence osteoclast formation through the MAPK pathway. This finding was further demonstrated by an *in vitro* cell culture study in which DHT inhibited the expression of osteoclast-associated genes as well as the activation of the MAPK pathway and the degradation of the nuclear factor kappa B (NF-κB) pathway.

In conclusion, TCM and its effective compounds show great promise for drug development. Potentially, natural compounds, such as genistein and DHT, may become alternative candidates for the treatment of bone and cartilage diseases. However, some important Research Topics need to be addressed. For example, the pharmacokinetic profiles of these botanicals still need to be elucidated and clinical trials should be undertaken. In addition, the underlying molecular mechanisms of natural compounds in treating bone and cartilage diseases may be complex due to their multi-target and multi-pathway characteristics. The interaction of natural compounds with the gut microbiome may also complicate pharmacological activity.
